# Correction: Mechanochemical activation of disulfide-based multifunctional polymers for theranostic drug release

**DOI:** 10.1039/d1sc90047a

**Published:** 2021-03-09

**Authors:** Zhiyuan Shi, Qingchuan Song, Robert Göstl, Andreas Herrmann

**Affiliations:** DWI – Leibniz Institute for Interactive Materials Forckenbeckstr. 50 52056 Aachen Germany goestl@dwi.rwth-aachen.de herrmann@dwi.rwth-aachen.de; Institute of Technical and Macromolecular Chemistry, RWTH Aachen University Worringerweg 1 52074 Aachen Germany; Zernike Institute for Advanced Materials, University of Groningen Nijenborgh 4 9747 AG Groningen The Netherlands

## Abstract

Correction for ‘Mechanochemical activation of disulfide-based multifunctional polymers for theranostic drug release’ by Zhiyuan Shi *et al.*, *Chem. Sci.*, 2021, **12**, 1668–1674, DOI: 10.1039/D0SC06054B.

Over the course of the publication of this article, ‘Mechanochemical activation of disulfide-based multifunctional polymers for theranostic drug release’, a citation to the related manuscript, ‘Mechanochemical bond scission for the activation of drugs’ by Shuaidong Huo *et al.*, could not be incorporated as it was not yet published.^1^ In the article by Shuaidong Huo *et al.*, we describe three independent systems that allow the activation and release of pharmaceutically active compounds by mechanical force. This article is an advancement of one of these systems. While we showed a related activation mechanism in the corresponding *Nat. Chem.* work, we describe in this article the unprecedented simultaneous release of pharmacologically active compounds alongside fluorophores for bioimaging, allowing the theranostic application of this mechanochemical principle and the direct monitoring of drug release. In addition we underline the universal character of the system by releasing multiple drugs alongside different fluorophores.

The Royal Society of Chemistry apologises for these errors and any consequent inconvenience to authors and readers.
